# Book Review

**Published:** 1947

**Authors:** 


### Reviews of Books

**Paediatrics for Nurses.** By Arthur G. Watkins, M.D., F.R.C.P. Pp. 192. Illustrated. Bristol: John Wright & Sons Ltd. 1947. Price 10s.—This is a very readable book with undoubtedly the correct style of description. The author has shown fine judgment in bringing together such of the useful information in paediatrics as might be considered adequate for nurses. The chapters are well defined and the subject matter admirably balanced; a sense of due proportion is maintained in considering the diseases which are more common and important. A tone of good practical standard is current throughout and the author's great clinical experience is often condensed into an admirably succinct sequence of sentences. Every paediatrician will note with satisfaction the emphasis laid on breast feeding, and this cannot be read too often, especially by those nurses who might be tempted, in busy domiciliary practice, from the best methods inculcated during their period of hospital training. Nurses will find a very helpful chapter on haemolytic disease and its relation to rhesus incompatibility. Since this book is intended primarily to cover descriptions of disease, the inclusion of excellent photographs and X-rays adds enormously to its value in this respect. It is a pleasure to see firmly expressed the view that good nursing is still the sheet-anchor in paediatric treatment. The book can be warmly recommended for its handy size, clear print, and good tables.

**Diseases of the Joints and Rheumatism.** By Kenneth Stone, D.M., M.R.C.P. Pp. ix., 362. Illustrated. London: William Heinemann (Medical Books) Ltd. 1947. Price 30s.—This book published in October of this year is excellently produced on a fine art paper, though the numerous photographs and illustrations are badly bound into the text. The book is, according to the Preface, written primarily for the senior medical student, yet the historical aspect of the conditions discussed will be of interest to even the most knowledgeable "rheumatologist." In fact, this useful review of early theories and writings is inclined to overshadow some of the more modern work. The extracts from the "classics," while making the book more easily readable and interesting, are inserted at the expense of recent research and theory. In rheumatoid arthritis, the pathology of nodules is well illustrated, but the more recent work on muscle biopsies carried out in America and more lately amplified and substantiated in this country, is omitted. The work of Talbot on heredity in gout and Copeman's theory of fatty nodules in fibrositis are not mentioned. The use of serial plasters in the correction of rheumatoid deformity is dealt with in a few lines and the value of a brace in spondylitis is curtly denied. Lactic acid or buffered phosphate injection into the joints in the treatment of osteoarthritis is, perhaps, new and unproven, but this technique is in such common use that a word on the subject would not have been out of place. The book is divided into two parts, one dealing with articular and the other with non-articular syndromes: as implied by the title, these are not all "rheumatic." They include such conditions as tuberculous disease of joints and rupture of the supraspinatus tendon. There is also a section on articular anatomy. In the second part, the author gives his own opinion on the inter-relationship of fibrositis, myalgia, vagotonia and hydration and one can see no reason for his diffidence and apologies for doing so. Earlier in the book he leans a little towards the virus theory in rheumatoid arthritis and states that "the inflamed joint of rheumatoid arthritis bears no resemblance whatever to any known allergic reaction"—perhaps a matter of opinion. This book has been carefully written and is very readable. The author's research into early bibliography and into the derivation of terminology is, perhaps, of more interest to the student of rheumatology than to the medical student.

**Cancer of the Breast.** By Duncan C. L. Fitzwilliams, C.M.G., M.D., Ch.M., F.R.C.S. Pp. vii., 199. Illustrated. London: William Heinemann (Medical Books) Ltd. 1947. Price 25s.—This book does not set out to do more than give the views of the author on certain aspects of breast carcinoma. He favours limited operations where practicable, with radium implantation, followed by radiotherapy. There is a good chapter on early diagnosis. He believes the flat-of-the-hand sign to be not only worthless but positively dangerous. Any local swelling of appreciable size can be felt with the flat of the hand but there are two swellings which cannot be so felt. The first is chronic mastitis, the second is a really early carcinoma. More than two hundred condensed case histories are included. These illustrate the author's arguments but the critical reader can hardly accept selected cases as supporting them. The same must be said of three full page photographs of patients showing cures following treatment. The book is well printed on good paper and is pleasant to handle and read, though the price seems rather high even judged by present-day standards. The author has clearly had very great experience in this disease. One feels that the material and the knowledge are available for something really worth while, but in its present form this book cannot be considered an important contribution to the literature on the subject.

**Materia Medica for Nurses.** By A. Muir Crawford, M.D., F.R.F.P.S.G. Sixth Edition. Pp. vi., 160. London: H. K. Lewis & Co. Ltd. 1947. Price 5s. 6d.—The sixth edition of this useful little book for nurses has just been published. It contains many important changes: especially in the sections dealing with sulphonamides, antiseptics, hypnotics and basal anaesthetics. These are matters upon which it is essential for nurses to be well-informed and up-to-date. Perhaps it would have been an improvement to have explained why those sulphonamides which are not rapidly absorbed into the blood have any therapeutic value. It is true they are recommended for use in intestinal infections and not in other conditions but a nurse might be excused if she wondered why.

The description of basal narcotics and the reasons for their employment in certain cases might, with advantage, have been a trifle expanded. The paragraph on page 53 goes some way towards explanation, but the mere list on pages 80 and 81 is a bald and unconvincing catalogue. The statement on page 117 that protamine zinc insulin is less likely to give rise to sharp and frequent attacks of hypoglycaemia is obscure, because the comparison is incomplete and leaves the reader asking "less likely than what?" Nevertheless this is a sound book for nurses to rely upon and the foregoing criticisms are made with further editions in view, not to give any impression of unreliability.

**Hey Groves' Synopsis of Surgery.** Edited by Sir C. P. G. Wakeley, K.B.E., C.B., D.Sc., F.R.C.S., F.R.S.E., F.A.C.S., F.R.A.C.S. Thirteenth Edition. Pp. viii., 637. Illustrated. Bristol: John Wright & Sons Ltd. 1947. Price 25s.—This little book is immensely popular and deservedly so. This edition has been given a considerable revision, and much new material added, to bring it up to date. But it embodies such an immense number of facts and opinions, compressed into so small a space, that for any one writer to give a true representation of modern surgery in a book of this size has become well nigh impossible. Events in the surgical world are moving fast, and printers and bookbinders are working slowly. That no doubt accounts for a number of omissions, such as the treatment of sacral bedsores by excision, of actinomycosis by penicillin (though it is mentioned in the section on penicillin), of curare in anaesthesia, of leucotomy in mental diseases, of transthoracic resection of neoplasms of the lower oesophagus, of Millin's retropubic prostatectomy, and of the scalene syndrome. The newer nomenclature of the four blood groups might well be given. We fear that the student in his finals who, relying on this synopsis, did not mention biopsy for the diagnosis of Hodgkin's disease, or ureteral catheterization to find out which is the tuberculous kidney, or mule-spinning as a cause of cancer of the scrotum, might fall on trouble. It is not clear whether orchidectomy ought or ought not to be done for malignant testis. The examiner might disagree with the advice to fix a movable kidney, or to do an immediate operation for pneumococcic peritonitis, or for acute pancreatitis, and he would be surprised to be told that appendicitis is four times as common in the male as in the female, or that it is a good scheme to do an appendicostomy to prevent recurrence of an intussusception. The only case which we have seen in which intussusception did recur was one in which this very thing had been done. Frankly, we think the time has come when venereal diseases, anaesthesia, orthopaedics, and ear, nose and throat surgery, should be delegated to specialists. For the general turn-out we have nothing but praise.

